# Combined Inoculation with Multiple Arbuscular Mycorrhizal Fungi Improves Growth, Nutrient Uptake and Photosynthesis in Cucumber Seedlings

**DOI:** 10.3389/fmicb.2017.02516

**Published:** 2017-12-19

**Authors:** Shuangchen Chen, Hongjiao Zhao, Chenchen Zou, Yongsheng Li, Yifei Chen, Zhonghong Wang, Yan Jiang, Airong Liu, Puyan Zhao, Mengmeng Wang, Golam J. Ahammed

**Affiliations:** ^1^College of Forestry, Henan University of Science and Technology, Luoyang, China; ^2^Department of Plant Science, Tibet Agriculture and Animal Husbandry College, Linzhi, China; ^3^College of Horticultural Science, Henan Agricultural University, Zhengzhou, China; ^4^College of Horticultural Science, South China Agricultural University, Guangzhou, China; ^5^Department of Horticulture, Zhejiang University, Hangzhou, China

**Keywords:** arbuscular mycorrhizal fungi, cucumber, photosynthesis, plant growth, nutrient uptake, RuBisCO

## Abstract

Mycorrhizal inoculation stimulates growth, photosynthesis and nutrient uptake in a wide range of host plants. However, the ultimate effects of arbuscular mycorrhyzal (AM) symbiosis vary with the plants and fungal species involved in the association. Therefore, identification of the appropriate combinations of AM fungi (AMF) that interact synergistically to improve their benefits is of high significance. Here, three AM fungal compositions namely VT (*Claroideoglomus* sp., *Funneliformis* sp., *Diversispora* sp., *Glomus* sp., and *Rhizophagus* sp.) and BF (*Glomus intraradices*, *G. microageregatum* BEG and *G. Claroideum* BEG 210), and *Funneliformis mosseae* (Fm) were investigated with respect to the growth, gas exchange parameters, enzymes activities in Calvin cycles and related gene expression in cucumber seedlings. The results showed that VT, BF and Fm could successfully colonize cucumber root to a different degree with the colonization rates 82.38, 74.65, and 70.32% at 46 days post inoculation, respectively. The plant height, stem diameter, dry weight, root to shoot ratio of cucumber seedlings inoculated with AMF increased significantly compared with the non-inoculated control. Moreover, AMF colonization greatly increased the root activity, chlorophyll content, net photosynthetic rate, light saturated rate of the CO_2_ assimilation (*A*sat), maximum carboxylation rate (*V*_cmax_) and maximum ribulose-1,5-bis-phosphate (RuBP) regeneration rate (*J*max), which were increased by 52.81, 30.75, 58.76, 47.00, 69.15, and 65.53% when inoculated with VT, respectively. The activities of some key enzymes such RuBP carboxylase/oxygenase (RuBisCO), D-fructose-1,6-bisphosphatase (FBPase), D-fructose-6-phosphatase (F6P) and ribulose-5-phosphate kinase (Ru5PK), and related gene expression involved in the Calvin cycle including *RCA*, *FBPase*, *FBPA*, *SBPase*, *rbcS* and *rbcL* were upregulated by AMF colonization. AMF inoculation also improved macro- and micro nutrient contents such as N, P, K, S, Ca, Cu, Fe, Mn, Mg, and Zn in roots. Further analysis revealed that inoculation with VT had relatively better effect on growth of cucumber seedling followed by BF and Fm, indicating that AMF composition consisting of distant AMF species may have a better effect than a single or closely related AMF spp. This study advances the understanding of plant responses to different AM fungi toward development of strategies on AMF-promoted vegetable production.

## Introduction

Arbuscular mycorrhizal fungi (AMF) are one of the most widely distributed species of endotrophic mycorrhizal fungi, belonging to a monophyletic phylum, the Glomeromycota that occurs in almost all terrestrial ecosystems (Wu et al., 2016). About 90% of the flowering plants, ferns and bryophytes can form a symbiotic association with AMF except for some *Brassicaceae* plants and plants from a few other families (Zhu et al., 2010; Liu et al., 2014).

Arbuscular mycorrhizal fungi are mainly composed of the hyphae, mycorrhiza and vacuole in root and the hyphae and spore in soil. They can form a huge hyphal network in the rhizosphere of plants, which not only promotes plant growth, yield and quality of vegetables (Bowles et al., 2016; Rozpadek et al., 2016), but also improves soil physical and chemical properties, and nutrients uptake from the soil (Chatzistathis et al., 2013; Baum et al., 2015). AMF improve nutritional status of plants by absorbing and translocating mineral nutrients beyond rhizospheric zone (Rouphael et al., 2015). Among the vegetable crops, species from *Solanaceae*, *Cucurbitaceae*, *Liliaceae* and some other families are all easy to form arbuscular mycorrhizal association with the exception of *Cruciferae*, *Chenopodiaceae*, and *Amaranthaceae*, which cannot or are less likely to be colonized with arbuscular mycorrhizae (Elbon and Whalen, 2015). AMF-induced positive effects on the growth and physiology have been reported in a range of plant species including kidney bean, pepper, watermelon, muskmelon, onion, tomato and asparagus (Beltrano et al., 2013; Baum et al., 2015; Rozpadek et al., 2016). We also found that tomato seedlings inoculated with *Funneliformis mosseae* exhibit significantly higher biomass, redox poise and calcium balance than non-AMF control plants (Liu et al., 2016). Proper selection of AMF and rhizobia that are compatible with each other as well as with the host plant can eventually increase the growth, yield and nutritional value of the legume crops including soybean (Meghvansi et al., 2008). Yadav et al. (2013) found that co-inoculation with *Acaulospora laevis* and *Glomus mosseae* on *Gloriosa superba* L. plantlets can interact synergistically and maximize benefits, resulting in higher leaf area, root length and colchicine content. In a previous study, we found that AMF inoculation could effectively improve growth of cucumber and other vegetable crops, which is closely associated with the secondary metabolism in plants (Liu et al., 2014).

Notably, different AMF alter growth and nutrient uptake of a given plant species differently (Ruscitti et al., 2017). Wang et al. (2008) demonstrated a positive stimulatory effect of *G. mosseae* on the growth of cucumber seedlings, whereas *G. versiforme* had an opposite effect. In addition, plant gene expression patterns in response to fungal colonization show a certain overlap when colonized with fungi of the *Glomeraceae* family. Earlier studies also showed that mycorrhizae-associated plant genes are differentially regulated in response to the different AM fungi (Feddermann et al., 2008). Therefore, it is necessary to identify the combination of AMF strains that interact synergistically to improve the benefits. It is well conceived that the application of AMF can enhance plant growth, photosynthesis and nutrient concentration, however, the underlying mechanisms still remain largely unknown. He et al. (2017) found differential effects of the three AMF species such as *Funneliformis mosseae* BEG167, *Rhizophagus intraradices* BEG141, and *Glomus versiforme* Berch on the two host plants (peanut and tomato) in terms of photosynthetic characteristics, growth and hormone status. Yang et al. (2014) also studied the effects of two AMF species, *Funneliformis mosseae* and *Rhizophagus intraradices* on plant growth, photosynthesis and nutrient concentration in black locust seedlings. Although they found significant improvement in the aforementioned parameters upon AMF colonization, no significant difference in the effect was noticed between AMF species. On the contrary, Zhu et al. (2014) found that *Glomus versiforme* had better effect than *Rhizophagus irregularis* in terms of the growth, gas exchange and chlorophyll fluorescence in black locust seedlings. These results imply that response of a plant species to AMF greatly vary depending on the AMF species. Therefore, we hypothesized that combination of multiple AMF species that are genetically distant may have different response compared to a combination that is genetically close. Thus, an attempt was made in the present study to explore the combination of AMF strains that interact synergistically to improve the benefits.

We systematically investigated the effects of three AMF compositions namely VT, which is a combination of multiple AMF spp from different genera such as *Claroideoglomus* sp., *Funneliformis* sp., *Diversispora* sp., *Glomus* sp. and *Rhizophagus* sp., BF, which is a combination of AMF from the same genera but different species such as *G. intraradices*, *G. microageregatum* BEG and *G. Claroideum* BEG 210., and *Funneliformis mosseae* (Fm), which is a single AMF species, on the growth, gas exchange parameters, Calvin cycles enzymes activities, related gene expression and nutrient concentration in cucumber seedlings. We found that VT had the greatest benefits followed by BF and Fm as evidenced by various morphological, physiological and molecular parameters. The results indicate that AMF composition consisting of different divergent AMF species may have a better effect than a single or closely related AMF spp. Thus, understanding the divergence in responses to different AM fungi is of great significance in developing strategies on AMF-promoted vegetable production.

## Materials and Methods

### Plant Culture, Mycorrhizal Inoculation and Treatments

Cucumber seeds (*Cucumis sativus* L. cv. Zhongnong No. 106) were soaked in 55°C warm water for 10 min, and germinated in a constant temperature oven at 30°C. AMF inocula used in this research were VT and BF provided by Symblom Company in Czech Republic. VT was composed of *Claroideoglomus* sp., *Funneliformis* sp., *Diversispora* sp., *Glomus* sp. and *Rhizophagus* sp. BF was composed of *G. intradices*, *G. microageregatum* BEG and *G. Claroideum* BEG 210. AMF inocula (*F. mosseae*, Fm) consisting of spores, soil, hyphae and infected clove (*Trifolium repens* L.) root fragment from a stock culture of *F. mosseae*, which were propagated by AMF inocula donated by Dr. Tunde Tackas, Hungarian Academy of Sciences. After 3 days, the germinated seeds were transplanted into 15 cm × 15 cm plastic pots containing 0.9 kg organic soil substrate (organic manure, soil and decomposed straw = 1:2:1). The chemical properties of the organic substrate that was sterilized for 4 h at 160°C were as follows: pH 7.43, 12.4% organic matter, 162 mg kg^-1^ available phosphorus, 473 mg kg^-1^ available nitrogen and 586 mg kg^-1^ available potassium The inoculation dosage was 10 g of inocula per pot containing about 2200 infective propagules/g in the inoculum as determined by MPN assay (Porter, 1979). Non-AM plants received the same weight of autoclaved inocula. Each experimental unit also received the same water extract from each AMF inoculant (obtained through a filtrate paper of 0.22 μm diameter) as controls to balance composition of the microbial community between inoculated and non-inoculated plants. The inocula were placed adjacent to roots of seedlings. The experimental pots were placed in solar greenhouse at an average temperature of 28°C/20°C (day/night) with photosynthetic photon flux density (PPFD) of 600 μmol m^-2^ s^-1^ and 85% relative humidity.

A complete randomized block design was used and thirty plants were arranged in each treatment consisting of three replicates. On 32, 39, 46, and 53 days after inoculation, plant height and stem diameter were measured by using a precision straight edge and Vernier caliper, respectively. Biomass, mycorrhizal infectivity, photosynthetic parameters, activity of enzymes involved in Calvin cycles and related gene expression were determined at 46 days after inoculation. Samples for various biochemical, physiological and gene expression analysis, unless otherwise stated, were immediately frozen in liquid nitrogen and stored at -80°C until analyses.

### Biomass, Photosynthetic Pigments and Root Activity Analyses

For dry biomass analysis, nine plants per treatment were randomly selected and divided into shoots and roots. Then they were dried in an oven at 80°C for 24 h and weighed to record their dry weights. Photosynthetic pigments were extracted from the fifth fully developed leaves in 80% acetone and the contents of chlorophyll a and chlorophyll b were measured colorimetrically (Lichtenthaler, 1987) by using a spectrophotometer (UV-1800, shimadzu, Japan). Root activity was determined by the triphenyl tetrazolium chloride (TTC) method (Comas et al., 2000). Briefly, fresh root tissues (0.5 g) were immersed in 10 ml 0.5 mM phosphate buffer solution containing 0.4% (w/v) TTC and kept in the dark at 37°C for 2 h. Then 2 ml 1 M H_2_SO_4_ was then added and the root was dried using filter papers and extracted with ethyl acetate. The extract which was red in color was collected in a volumetric flask and the volume was made 10 ml by adding ethyl acetate. The absorbance of the red extract was recorded at 485 nm. Root activity was calculated using following equation: Root activity = amount of TTC reduction (μg)/fresh root weight (g) × time (h).

### Root Colonization and Mycorrhizal Infectivity

A fraction of the roots (0.5 g) were carefully washed and cut into 1-cm long segments. Root segments were cleared with 10% KOH at 90°C for 20 min and rinsed with water prior to acidification with 2% HCl for 5 min. Then the roots segments were stained with 0.01% acid fuchsin (Kormanik et al., 1980). Mycorrhizal colonization rate was measured using the gridline intercept method described by Giovannetti and Mosse (1980) at 46 days after inoculation.

### Nutrient Analysis

Roots (0.3 g dry mass) were pre-digested with a mixture of 5 ml concentrated HNO_3_ and 2 ml H_2_O_2_ (30%) overnight at room temperature followed by microwave heating for 15 min as described previously (Wu et al., 1997). Afterward the digestates were diluted to a final solution contained 2% HNO_3_. The contents of nutrients, potassium (K), phosphorus (P), calcium (Ca), copper (Cu), iron (Fe), manganese (Mn), magnesium (Mg), zinc (Zn), and sulfur (S) were determined by injecting digested samples into an inductively coupled plasma mass spectrometer (ICP-MS, Agilent 7500ce, Agilent Technologies, United States). Total nitrogen content was determined using the Kjeldahl method (Hanon K9840 Kjeldahl apparatus), as described by Yan et al. (2012).

### Gas Exchange Measurements

Gas exchange parameters such as net photosynthetic rate (Pn), stomatal conductance (Gs) and intercellular CO_2_ concentration (Ci) were measured using an infrared gas analyzer based portable photosynthesis system (LI-6400; LI-COR, Lincoln, NE, United States) on the fifth leaf of each plant at 46 days after inoculation. The light saturated rate of the CO_2_ assimilation (*A*sat) was measured at ambient CO_2_ concentration of 360 μmol mol^-1^ and saturating photosynthetic photon flux density (PPFD, 1000 μmol m^-2^ s^-1^) with a leaf temperature of 25 ± 1.5°C and air relative humidity of 80–90%. Assimilation versus intercellular CO_2_ concentration (*A*/*C*i) curves were measured according to von Caemmerer and Farquhar (1981). The maximum Rubisco carboxylation rates (*V*c, max) and maximum RuBP regeneration rates (*J*max) were estimated from the *A*/*C*i curves using the method of Ethier and Livingston (2004).

### Determination of Enzymes Activity Involved in Calvin Cycle

Ribulose-1,5-bis-phosphate (RuBP) carboxylase/oxygenase (RuBisCO) activity was measured spectrophotometrically by coupling 3-phosphoglyceric acid formation with NADH oxidation at 25°C, following the method described by Lilley and Walker (1974). For measurements of RuBisCO activity, frozen leaf samples were ground to fine powder in liquid N_2_ and then extracted in a solution containing 50 m*M* Tris-HCl (pH 7.5), 1 m*M* EDTA, 1 m*M* MgCl_2_, 12.5% (v/v) glycerin, 10% PVP, and 10 m*M* β-mercaptoethanol. The homogenate was centrifuged at 15,000 *g* for 15 min at 4°C. Total Rubisco activity was assayed after the crude extract was activated in a 0.1 ml activation solution containing 33 m*M* Tris-HCl (pH 7.5), 0.67 m*M* EDTA, 33 m*M* MgCl_2_, 10 m*M* NaHCO_3_ for 15 min. Initial Rubisco activity was measured by coupling the activity to NADH oxidation using PGA kinase and GAP dehydrogenase as previously described (Sharkey et al., 1991). The oxidation of NADH was followed by changes in absorbance at 340 nm for 90 s.

Ribulose-5-phosphate kinase (Ru5PK; EC 2.7.1.19) was measured using the same protocol for Rubisco, however, the reaction was started with ribulose-5-phosphate (0.5 mM). PGA was determined as described by Usuda (1985). The reaction mixture contained: 40 mm HepesKOH (pH 7.8), 5 mM ATP, 0.2 mM NADH, 5 mM phosphocreatine, 10 units/mL of creatine phosphokinase (EC 2.7.3.2), 5 units/mL of NAD-G3P dehydrogenase (EC 1.2.1.12), and 5 units/mL of PGA-kinase (EC 2.7.2.3). The reaction was initiated by the addition of an aliquot of sample. FBPase activity was determined by monitoring the increase in A340 using an extinction coefficient of 6.2 m*M*^-1^ cm^-1^ (Scheibe et al., 1986; Zhou et al., 2007). Initial activity was assayed immediately after homogenization. Total activity was assayed on aliquots of enzyme extract incubated for 20 min with 100 m*M* dithiothreitol, 2 m*M* Fru-1,6-bisP, 10 m*M* MgCl_2_, and 0.1 *M* HEPES-NaOH (pH 8.0). The assay mixture for initial and total activities, maintained at 25°C, consisted of 0.1 *M* HEPES-NaOH (pH 8.0), containing 0.5 m*M* Na_2_EDTA, 10 m*M* MgCl_2_, 0.3 m*M* NADP^+^, 0.6 m*M* Fru-1, 6-*bis*P, 0.6 U Glc-6-P dehydrogenase from bakers’ yeast (Sigma–Aldrich, China), 1.2 U Glc-P-isomerase from bakers’ yeast (Sigma–Aldrich, China), and 100 μl of enzyme extract in a final volume of 1 ml. The reaction was initiated by the addition of enzyme extract.

### Determination of Transcript Abundance

Total RNA was isolated from young leaves of both mycorrhizal and non-mycorrhizal plants using Trizol reagent (Sangon, China) according to the manufacturer’s instruction. Genomic DNA was removed with RNeasy Mini Kit (Qiagen, Germany). Total RNA (1 μg) was reverse-transcribed using ReverTra Ace qPCR RT Kit (Toyobo, Japan) following the manufacturer’s instruction. Quantitative real-time PCR was performed using the iCycler iQ^TM^ real-time PCR detection system (Bio-Rad, Hercules, CA, United States). Each reaction (25 μL) consists of 12.5 μL SYBR Green PCR Master Mix (Takara, Japan), 1 μl of diluted cDNA and 0.1 μmol of forward and reserve primers. PCR cycling conditions were as follows: 95°C for 3 min, followed by 40 cycles of denaturation at 95°C for 30 s, annealing at 58°C for 30 s and extension at 72°C for 30 s. *Actin* of cucumber (GenBank AB698859.1) was used as an internal standard. The gene-specific primers used for the amplification were determined on the basis of gene or EST sequences and are listed in Supplementary Table S1. The quantification of mRNA levels is based on the method of Livak and Schmittgen (2001).

### Statistical Analysis

All data presented for different biochemical and physiological analysis are averages of at least four repetitions of each treatment. Data were statistically analyzed using one-way analysis of variance (AVONA), and tested for significant (*P* ≤ 0.05) treatment differences using Tukey′s test. The data were plotted using Origin 7.0 software (OriginLab, Northampton, MA, United States).

## Results

### Effects of AMF on Plant Height and Stem Diameter of Cucumber Seedlings

**FIGURE 1:**
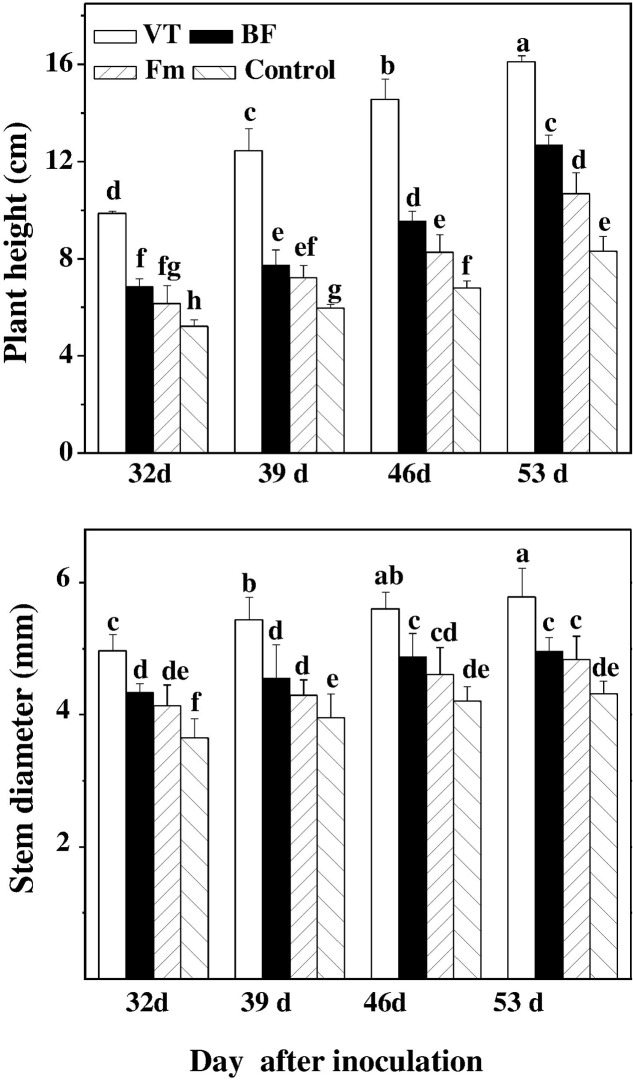
Effect of AMF colonization on plant height and stem diameter of cucumber seedlings. On 32, 39, 46, and 53 days after inoculation, plant height and stem diameter were measured by using a precision straight edge and Vernier caliper, respectively. Means denoted by the same letter did not significantly differ at *P* ≤ 0.05, according to Tukey’s test. VT was composed of *Claroideoglomus* sp., *Funneliformis* sp., *Diversispora* sp., *Glomus* sp. and *Rhizophagus* sp. BF were composed of *G. intradices*, *G. microageregatum* BEG, *G. Claroideum* BEG 210. AMF inocula (*F. mosseae*, Fm) consisting of spores, soil, hyphae and infected clove (*Trifolium repens* L.) root fragment from a stock culture of *F. mosseae*.

The growth indices, such as plant height and stem diameter of cucumber seedlings were investigated at 32, 39, 46, and 53 days after inoculation with AMF. The plant height and stem diameter of AMF-inoculated seedlings (especially those with VT) significantly improved as compared to that of the control during the tested time points. And there was a rapid increase with the progress of AMF colonization. In comparison to the non-inoculated control, the plant height and stem diameter of cucumber seedlings inoculated with VT increased by 55.79 and 33.80% on day 53, followed by BF and Fm, which increased plant height/stem diameter by 26.02%/14.81% and 22.46%/11.81%, respectively on day 53 (**Figure 1**).

### Effects of Different AMF Strains on Biomass Production and Mycorrhizal Colonization Rate of Cucumber Seedlings

As shown in **Table 1**, the growth of cucumber seedlings was significantly improved by inoculation with AMF. For instance, compared to the control, VT inoculation increased dry weight of shoot, dry weight of root and root to shoot ratio of dry weight by 72.15, 112.94, and 23.72%, respectively, while Fm inoculation increased those by 27.24, 58.22, and 24.51%, respectively, implying that AMF-induced growth increment was predominantly attributed to root as compared to shoot. Moreover, the infection rates of cucumber seedlings inoculated with VT, BF and Fm were 82.38, 74.65, and 70.32% on day 46 after inoculation. The results demonstrated that inoculation with AMF effectively promoted biomass production in cucumber seedlings.

**Table 1:** Effects of VT, BF, and Fm on growth and colonization percentage in cucumber seedlings.

Treatments	Shoot DW (g plant^-1^)	Root DW (g plant^-1^)	Root-shoot ratio	AMF colonization (%)
VT	6.206 ± 0.241 a	1.942 ± 0.156 a	0.313 ± 0.018 a	82.38 ± 3.56 a
BF	4.694 ± 0.569 b	1.456 ± 0.213 b	0.310 ± 0.014 b	74.65 ± 5.64 b
Fm	4.587 ± 0.356 b	1.443 ± 0.107 b	0.315 ± 0.021 b	70.32 ± 7.42 b
Control	3.605 ± 0.407 c	0.912 ± 0.12 c	0.253 ± 0.035 c	–

*All data presented are averages of four repetitions of each treatment. Means denoted by the same letter did not significantly differ at P ≤ 0.05, according to Tukey’s test. Biomass was determined on 46 days after inoculation.*

### Effects of Different AMF Strains on Root Activity and Chlorophyll Contents in Cucumber Seedlings

To further investigate the impact of AMF colonization on cucumber seedlings, we determined root activity and chlorophyll contents. A significant enhancement in the contents of chlorophyll a, chlorophyll b and total chlorophyll (Chla + Chlb) was observed in AMF-inoculated seedlings accompanied with increased root activity (**Table 2**). Compared with non-AMF control, the total chlorophyll content and root activity in VT-inoculated cucumber seedlings increased by 30.75 and 52.81%, respectively. Meanwhile, the total chlorophyll content and root activity of Fm-inoculated cucumber seedlings increased by 17.47 and 24.91%, respectively.

**Table 2:** Effects of VT, BF and Fm on chlorophyll contents and root activity in cucumber seedlings.

Treatments	Chlorophyll a (mg.g^-1^ FW)	Chlorophyll b (mg.g^-1^ FW)	Total chlorophyll (mg.g^-1^ FW)	Root activity μg.g^-1^.FW h^-1^
VT	3.36 ± 0.23 a	1.13 ± 0.11 a	4.49 ± 0.34 a	57.84 ± 3.46 a
BF	3.15 ± 0.28 b	0.97 ± 0.09 b	4.12 ± 0.25 b	49.72 ± 3.78 b
Fm	3.08 ± 0.12 b	0.96 ± 0.06 b	4.03 ± 0.27 b	47.28 ± 7.42 b
Control	2.61 ± 0.25 c	0.82 ± 0.06 c	3.43 ± 0.28 c	37.85 ± 5.69 c

*Data are means of four replicates. Means denoted by the same letter did not significantly differ at P ≤ 0.05, according to Tukey’s test. Chlorophyll contents and root activity were determined on 46 days after inoculation.*

### Effects of AMF Strains on Nutrient Content in Cucumber Seedlings

As shown in **Table 3**, AMF colonization stimulated nutrient uptake in the roots of AMF-inoculated plants. The contents of N, P, K, Ca, Cu, Fe, Mn, Mg, Zn, and S increased by 91.16, 33.47, 92.38, 86.85, 50.95, 30.16, 112.50, 73.68, 61.44, and 99.28%, respectively in cucumber seedlings inoculated with VT as compared to that of non-AMF control. Although the nutrient contents in BF- and Fm- inoculated plants were higher than that in control, those were all lower than that of VT-inoculated plants.

**Table 3:** Effects of VT, BF, and Fm on nutrient contents in cucumber seedlings.

Treatments	N (g/kg)	P (g/kg)	K (g/kg)	Ca (g/kg)	Cu (mg/kg)	Fe (mg/kg)	Mn (mg/kg)	Mg (mg/kg)	Zn (mg/kg)	S (mg/kg)
VT	53.18 ± 4.2a	6.42 ± 0.5a	35.84 ± 3.6a	44.19 ± 4.5a	41.45 ± 2.8a	0.82 ± 0.05a	0.17 ± 0.02a	12.87 ± 1.5a	12.98 ± 1.2a	5.54 ± 0.3a
BF	49.29 ± 3.9ab	5.93 ± 0.4ab	25.68 ± 2.7b	37.58 ± 3.3b	37.21 ± 2.5b	0.66 ± 0.04bc	0.12 ± 0.01b	10.24 ± 1.2b	11.04 ± 1.1b	4.93 ± 0.2b
Fm	48.63 ± 3.2b	5.43 ± 0.5b	24.64 ± 3.1b	35.74 ± 3.8b	35.12 ± 3.7b	0.71 ± 0.06b	0.15 ± 0.03ab	9.03 ± 0.9c	10.86 ± 1.0b	4.86 ± 0.2b
Control	27.82 ± 5.5c	4.81 ± 0.4c	18.63 ± 2.2c	23.65 ± 4.2c	27.46 ± 3.1c	0.63 ± 0.04c	0.08 ± 0.01c	7.41 ± 0.8d	8.04 ± 0.9c	2.78 ± 0.3c

*Data are means of four replicates. Means denoted by the same letter did not significantly differ at P ≤ 0.05, according to Tukey’s test. Nutrient contents were determined on 53 days after inoculation.*

### Effects of AMF Strains on Gas Exchange Parameters of Cucumber Seedlings

**FIGURE 2:**
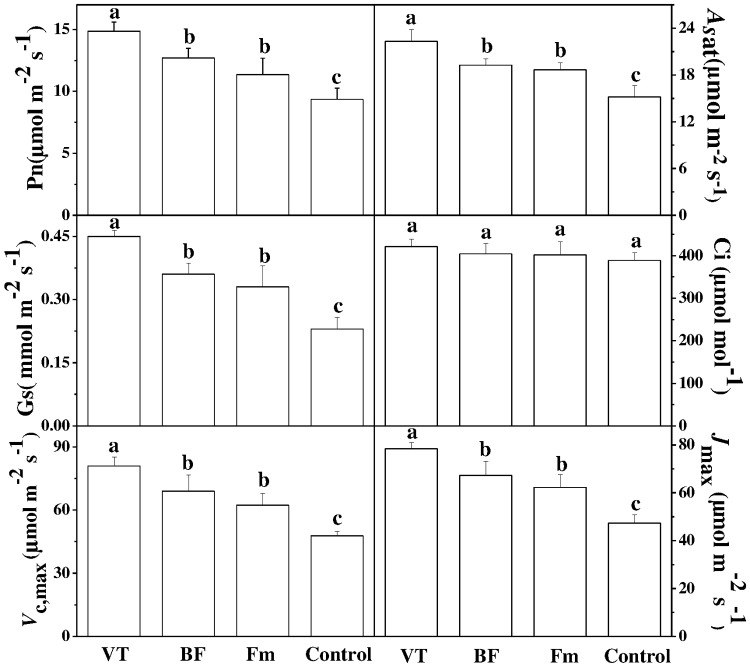
Effect of AMF inoculation on gas exchange parameters in cucumber seedlings. Gas exchange parameters such as net photosynthetic rate (Pn), stomatal conductance (Gs) and intercellular CO_2_ concentration (Ci) were measured using an infrared gas analyzer based portable photosynthesis system (LI-6400; LI-COR Lincoln NE, USA) on the fifth leaf of each plant at 46 days after inoculation. *A*sat, light-saturated net assimilation rate; *V*c, *max*, maximum carboxylation rate of Rubisco; *J*max, maximum RuBP regeneration rates. Data are the means of 10 replicates with SDs shown by vertical bars.

To determine the photosynthetic response of cucumber seedlings to different AMF strains, some key gas exchange parameters were examined. Compared with the non-AMF control, the net photosynthetic rate (*P*n) and stomatal conductance (*G*s) of VT-inoculated seedlings increased significantly by 58.76 and 95.65%, respectively. Intriguingly, similar trend was also found in BF-inoculated plants as compared to the non-inoculated control, specifically, the *P*n of seedlings inoculated with BF and Fm increased by 35.79 and 21.26%, respectively (**Figure 2**). However, no significant difference in intercellular CO_2_ concentration (*C*i) was observed between the AMF-inoculated plants and the controls. Furthermore, AMF inoculation induced a significant increase in *A*sat, *V*cmax, *J*max values in the leaves of cucumber seedlings as compared to that of non-inoculated control. Although the values of *A*sat, *V*cmax, *J*max of BF-inoculated and Fm -inoculated plants increased, those were lower than that of VT-inoculated plants.

### Effects of AMF Strains on Related Enzyme Capacities and Expression of Calvin Cycle Genes

**FIGURE 3:**
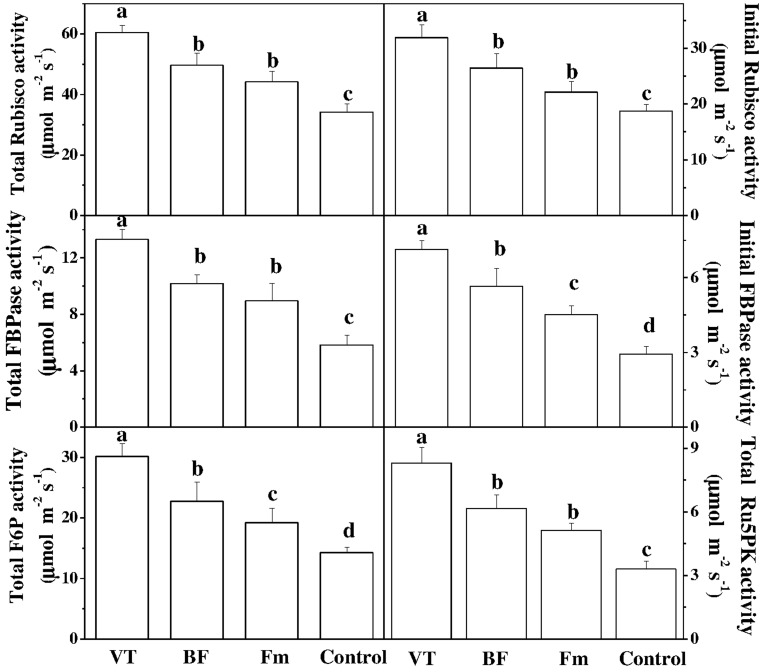
Effect of AMF inoculation on the activity of enzymes involved in the Calvin cycle. Data are the means of four replicates with SDs shown by vertical bars. Means followed by the same letter are not significantly different according to Tukey’s test (*P* < 0.05). RuBisCO, ribulose-1,5-bis-phosphate carboxylase/oxygenase; FBPase, D-fructose-1,6-bisphosphatase; F6P, D-fructose-6-phosphatase; Ru5PK- ribulose-5-phosphate kinase.

To get a better insight into the mechanism of AMF-regulated enhancement in photosynthesis, we analyzed some key enzymes activities of the Calvin cycle. The results showed that AMF inoculation significantly induced the total and initial activities of ribulose-1,5-bisphosphate carboxylase/oxygenase (RuBisCO), by 76.63 and 70.28% in VT-inoculated plants as compared to non-AMF control, respectively (**Figure 3**). Similar trends were found for several other enzymes including total and initial FBPase activities, Ru5PK and PGA in leaves of AMF-inoculated plants, which were also substantially higher than that of mock plants (**Figure 3**). These results strongly suggest that AMF promoted photosynthesis by positively regulating Calvin cycle enzymes. Moreover, an increased enzyme activity was observed in VT-inoculated plants as compared to that in BF as well as Fm plants. This implies that the induction of CO_2_ assimilation in AMF seedlings was accompanied by increased activities of key enzymes involved in CO_2_ assimilation.

**FIGURE 4:**
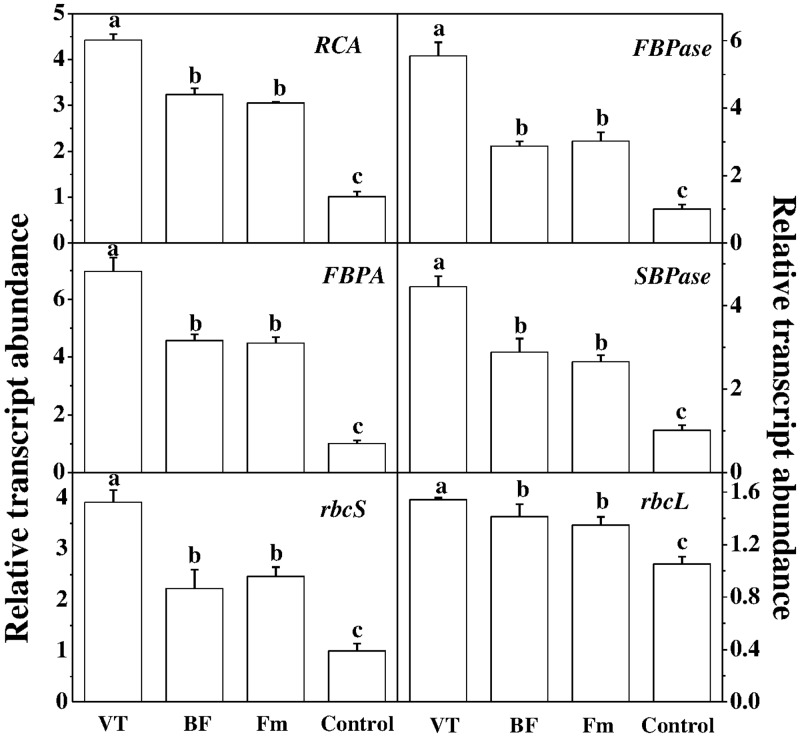
Effect of AMF colonization on gene expression involved in the Calvin cycle. The expression of genes was analyzed by quantitative RT-PCR using gene-specific primer pairs (Supplementary Table S1). Data are the means of four replicates with SDs shown by vertical bars. Means followed by the same letter are not significantly different according to Tukey’s test (*P* < 0.05).

To further investigate how AM fungi affected photosynthesis, we analyzed transcript levels of six Calvin-Benson cycle genes (**Figure 4**), encoding ribulose-1,5-bisphosphate carboxylase/oxygenase activase (*RCA*), ribulose-1,5-bisphosphate carboxylase/ oxygenase small subunit (*rbcS*), ribulose-1, 5-bisphosphate carboxylase large subunit (*rbcL*), fructose-1,6-bisphosphatase (*FBPase*), sedoheptulose-1,7-bisphosphatase(*SBPase*) and fructose-1,6-bisphosphate aldolase (*FBPA*) in leaves. As shown in **Figure 4**, compared with the control, transcript levels of *RCA*, *FBPase*, *FBPA*, *SBPase* and *rbcS* increased by 3.37, 4.48, 5.95, 3.41, and 2.90 folds in VT inoculated plant leaves, while Fm inoculation increased those transcripts by 2.01, 1.99, 3.47, 1.63, and 1.45 folds, respectively.

## Discussion

The universal association between AMF and plants is such an old tie that, perhaps, enabled the establishment of plants in land (Rouphael et al., 2015). Over the last couple of decades, AMF have been implicated in boosting plant growth, photosynthesis, nutrient acquisition and tolerance to biotic and abiotic stresses (Liu et al., 2014, 2016; Cavagnaro et al., 2015). However, use of appropriate AMF, either individually or in combination, still remains a big challenge as the benefits from the mutualism greatly vary depending on the AMF strains. In this study, we used combinations of multiple or single species of AMF strains to investigate their effects on plant growth, photosynthesis and nutrient uptake in cucumber plants. We found that plant height, dry weight of shoot and root, root to shoot ratio, root activity, chlorophyll content, photosynthetic characteristics and nutrient contents in cucumber seedlings inoculated with AMF were significantly higher than those of the control. We also noticed that VT, which is a combination of multiple AMF spp from different genera such as *Claroideoglomus* sp., *Funneliformis* sp., *Diversispora* sp., *Glomus* sp., and *Rhizophagus* sp. had better effect on cucumber growth as compared to that of BF, which is a combination of AMF from the same genus but different species such as *G. intradices*, *G. microageregatum* BEG and *G. claroideum* BEG 210. Additionally, VT and BF both appeared to be more effective than Fm, which is a single AMF species, *Funneliformis mosseae*. These results were in agreement with the previous findings that AMF colonization could promote the synthesis of chlorophyll and carotenoid, increase the root absorption area and root activity, strengthen the absorption and transport of water and other nutrients and/or mineral elements such as P, K, Mg, and Mn, thereby enhancing the photosynthesis and biomass accumulation in plants (Baslam et al., 2013).

Plant growth parameters such as plant height, stem diameter and biomass production are the external indicators of internal plant metabolism. In the present study, plant height and stem diameter of mycorrhizal seedlings remained consistently higher than those of non-mycorrhizal seedlings throughout the study period (**Figure 1**). These data are in accord with Yang et al. (2014), who found significantly increased plant height and stem diameter upon inoculation with *Funneliformis mosseae* or *Rhizophagus intraradices* in black locust plants. In addition, AMF colonization increased the root to shoot ratio in AMF-colonized plants, indicating that AMF inoculation, perhaps, further increased nutrient absorption area in AMF-colonized plants. It is to be noted that biomass accumulation is largely determined by the photosynthetic performance of a plant. Since AMF inoculation enhanced both nutrient uptake and photosynthesis in cucumber plants (**Figure 2** and **Table 3**), it is highly likely that AMF-induced increased synthesis of photosynthates contributed to both below-ground and aboveground biomass accumulation. Furthermore, we noticed a significantly higher chlorophyll content in AMF-inoculated plants, which was accompanied with an increased N status in the roots. Since chlorophyll molecules trap N, AMF-induced enhanced N uptake might contribute to higher chlorophyll contents in the AMF-inoculated plants (de Andrade et al., 2015). Moreover, increased chlorophyll contents in mycorhizal plants can also be associated with increased P and Mg uptake (Zhu et al., 2014).

Notably, a potential influence of AMF in strengthening carbon sink may trigger photosynthesis in the host plants. When plants are colonized by AMF, roots appear to be the strongest sink for carbohydrates since fungi can utilize 20% of the photosynthates produced by host plants (Porcel et al., 2015). Thus, AMF remarkably alter source-sink relationships by stimulating the exchange of carbohydrates and mineral nutrients. Kaschuk et al. (2009) infer that stimulation in Calvin cycle by AMF can boost export of triose P to the root that reduces supposed limitation on photosynthesis, leading to the enhancement in the rate of CO_2_ fixation. In the current study, AMF colonization increased Pn, Asat and Gs, but did not alter Ci (**Figure 2**). As Gs and chlorophyll content increased in mycorrhizal plants, both stomatal and non-stomatal factors were involved in the AMF-induced enhancement in the photosynthesis. AMF colonization was able to improve the gas exchange capacity of the cucumber plants most likely by maintaining stomatal opening, reducing stomatal resistances and increasing transpiration fluxes. As observed by Porcel et al. (2015) in rice, AMF-induced increase in photochemical efficiency for CO_2_ fixation and solar energy utilization can eventually improve biomass production in plants.

The rate of photosynthesis is under strict control of two main biochemical processes known as ribulose-1,5-bis-phosphate (RuBP) carboxylase/oxygenase (RuBisCO) carboxylation and RuBP regeneration (Amthor, 1995). In general, RuBisCO activity positively influences CO_2_ assimilation rate in plants. In the current study, AMF colonization increased *V*_cmax_ and *J*_max_, indicating that AMF promoted photosynthesis by increasing both RuBisCO carboxylation and regeneration capacity in mycorrhizal plants. In addition to the measurement of RuBisCO activity from CO_2_ response curves with an infrared gas analyzer (IRGA), we performed biochemical assay that determined direct enzymatic activity of RuBisCO. We found that AMF inoculation significantly induced some key enzymes activities of the Calvin cycle including RuBisCO, FBPase and F6P etc. Activation of RuBisCO would induce *V*_cmax_, while an increment in *J*_max_ is associated with the improvement of key regulatory enzymes such as sedoheptulose-1,7-bisphosphatase (SBPase) and D-fructose-1,6-bisphosphatase (FBPase) in the Calvin cycle (Ölcer et al., 2001). FBPase catalyzes the hydrolysis of D-fructose-1,6-bisphosphate (FBP) to D-fructose-6-phosphate (F6P) and orthophosphate, and is a key enzyme in gluconeogenesis (Mano et al., 2009), whereas SBPase is a critical enzyme involved in photosynthetic carbon fixation in the Calvin cycle (Raines et al., 1999). In our study, RuBisCO activity was well correlated with the expression of genes encoding for small (rbcS) and large (rcbL) RuBisCO subunits, indicating that AMF inoculation affects transcriptional process leading to the enhancement in photosynthesis. Additionally, RuBisCO activation depends on the activity of RuBisCO activase (RCA) (Ji et al., 2015). In accordance with the enzymes activities in Calvin cycle, AMF inoculation increased expression levels of *RCA*, *FBPase*, *FBPA*, *SBPase*, *rbcS* and *rbcL*. The results suggest that AMF invoked a range of related enzymes and genes encoding Calvin cycle key enzymes to facilitate photosynthetic metabolism.

In the present study, we found that AMF inoculation improved nutrient uptake including N, P, K, Ca, Cu, Fe, Mn, Mg, Zn, and S, which are in agreement with the previous reports (Wang et al., 2008; Ortas et al., 2011). Most strikingly, previous studies suggested that dual AMF species inoculation could increase concentrations of Cu, Mg and rutin (Zubek et al., 2015). Bona et al. (2015) also reported that strawberry ‘Selva’ inoculated with a commercial AMF containing *R. intraradices*, *G. ageratum*, *G. viscosum*, *C. etunicatum*, and *C. claroideum* with 70% of the conventional fertilization had higher yield, fruit number, and larger size of the fruits than non-inoculated plants with conventional fertilization. The present results support this view and suggest that VT-inoculation is more effective for augmenting plant mass and nutrient contents. Our observations are contrary to Janoušková et al. (2009), who found that dual inoculation with *R. intraradices and C. claroideum* did not result in any additional benefit to host plants in comparison with single inoculation. Moreover, plant growth depression caused by *C. claroideum* also persisted in the mixed treatment. By contrast, Jansa et al. (2008) found that leek colonized by a mixture of *R. intraradices* and *C. claroideum* acquired more P than with either of the two isolates separately. Since we used almost equal amount of inocula for mycorhyzal colonization of VF, BF and Fm, it is quite possible that AMF composition containing divergent species may have a better effect than a single or closely related AMF spp. Moreover, VT had the highest root colonization rate and provided the maximum benefits followed by BF and Fm. This could be due to the highest arbuscule abundance in VT, which might function in improving plant growth and nutritional status. However, the in-depth mechanisms still remain to be investigated using physiological and quantitative molecular tools.

## Conclusions

The present results showed that the AM symbiosis could enhance the growth, biomass, root activity, nutrient content and gas exchange parameters in AMF-inoculated plants. Moreover, we propose that inoculation with VT has better growth-promoting effect on cucumber seedlings, as evidenced by a higher CO_2_ assimilation, gas exchange parameters and key enzymes activities and gene expression of the Calvin cycle. Our results also suggest that incorporation of multiple mycorrhizal fungi that are genetically distant into commercial nursery system could induce higher photosynthetic ability and nutrient uptake in AM plants, thus leading to improved plant biomass production. However, the mechanism of interaction among the combination of AMF strains would require further exploration. Since global food security is a big challenge of 21st century, utilization of AMF can be an environmentally sustainable option for the enhancement of global food production (Cavagnaro et al., 2015).

## Author Contributions

AL and SC conceived and designed the research. SC, CZ, HZ, YJ, YC, and MW performed the experiments and analyzed the data. YL and ZW supervised the study. SC, GA, PZ, and AL wrote the manuscript. All authors read and approved the final manuscript.

## Conflict of Interest Statement

The authors declare that the research was conducted in the absence of any commercial or financial relationships that could be construed as a potential conflict of interest.
