# Supplementary material for: Combined Inoculation with Multiple Arbuscular Mycorrhizal Fungi Improves Growth, Nutrient Uptake and Photosynthesis in Cucumber Seedlings
Source: Front Microbiol. 2017 Dec 19;8:2516. doi: 10.3389/fmicb.2017.02516 (PMC5742139; doi:10.3389/fmicb.2017.02516)
Supplement: Supplementary file 1 [file Table_1.DOCX]

**Supplementary Table S_1_** Genes and primers used in quantitative real-time PCR analysis

| Gene | Accession no. | Forward primer | Reverse primer |
| --- | --- | --- | --- |
| *RCA* | XM_011657999.1 | 5’- CGATGAAGTGAGAAAGTGGGCTGT-3’ | 5’- TCCATGACGAGCATGTTGCC-3’ |
| *FBPase* | XM_004140842.2 | 5’- TTCACAGCGCCATGGATCAC-3’ | 5’- GCAGACGAACTTGCAGCCAA-3’ |
| *FBPA* | XM_004139723.2 | 5’- GTGGACAAGGGAACCGTGGA-3’ | 5’- CGGTAGCAGCAGCACACTTG-3’ |
| *SBPase* | FJ911553.1 | 5’- GACATGGGAGGCCCTGTTGA-3’ | 5’- GCGAGGACCGTAAACTCCCA-3’ |
| *rbcS* | XM_004135046.2 | 5’- ACAGTGCAATGCCTCTCAGACT-3’ | 5’- GTGGCCACACCTTCATGCAC-3’ |
| *rbcL* | KT779307.1 | 5’- AGACCGAAGCCGCGGTATTT-3’ | 5’- CCCAAAGTTCCTCCGCCGAA-3’ |
| *actin* | AB698859.1 | 5’-TGGACTCTGGTGATGGTGTTA-3’ | 5’-CAATGAGGGATGGCTGGAAAA-3’ |
